# What do hospital-based health professionals need and expect from an mHealth app to support the first 1000 days of life? Results from a cross sectional study

**DOI:** 10.1371/journal.pone.0284448

**Published:** 2023-04-21

**Authors:** Laura Brunelli, Raffaella Dobrina, Chiara De Vita, Elena Mazzolini, Giuseppa Verardi, Sofia Bussolaro, Maura Degrassi, Giulia Hervatich, Maria Piazza, Tamara Strajn, Andrea Cassone, Anja Starec, Giuseppe Ricci, Sara Zanchiello, Tamara Stampalija

**Affiliations:** 1 Department of Medical, Surgical and Health Sciences, University of Trieste, Trieste, Italy; 2 Healthcare Professions Department, Institute for Maternal and Child Health – IRCCS “Burlo Garofolo”, Trieste, Italy; 3 Area Science Park, Trieste, Italy; 4 Department of Epidemiology, Istituto Zooprofilattico Sperimentale delle Venezie, Udine e Legnaro, Italy; 5 Obstetrics and Gynecology Clinic, Institute for Maternal and Child Health – IRCCS “Burlo Garofolo”, Trieste, Italy; 6 Unit of Fetal Medicine and Prenatal Diagnosis, Institute for Maternal and Child Health - IRCCS "Burlo Garofolo", Trieste, Italy; Duervation, AUSTRIA

## Abstract

**Background:**

Several attempts have been made to introduce mHealth solutions to support maternal, newborn, and child health (MNCH). However, most of the available apps do not meet the needs of end-users, underscoring the urgency of involving them in the co-design of telehealth interventions.

**Objective:**

With this in mind, we investigated the needs and expectations of hospital-based health professionals (i.e., secondary users) providing care to pregnant women and new mothers with their babies for a hypothetical mHealth app to support the first 1000 days of life.

**Methods:**

From November 2021 to March 2022, we surveyed health professionals using a questionnaire that explored the perceived importance of specific content, functionalities, and technical features of the proposed app. We also collected sociodemographic information from secondary users. We performed descriptive analysis and then used Ward hierarchical clustering method to classify respondents according to their response patterns.

**Results:**

We recorded the needs and expectations of 145 hospital-based health professionals from obstetrics/gynecology, nursery/neonatology, and pediatrics. We found general agreement with the proposed content of the app, particularly general information about health during pregnancy (92%) and potential risky infections during pregnancy (91%). Three clusters emerged from the analysis, with the high and medium demanding clusters rating the importance of app content and technical features as very high and high, respectively, while low demanding cluster expressing more skepticism, especially about some of the proposed functionalities of the app.

**Conclusions:**

Assessing the needs and expectations of end-users is an essential process for developing tailored and effective mHealth solutions. This study has shown that hospital-based health professionals generally recognize the value of the proposed app, suggesting their propensity to integrate such a telehealth solution into mainstream clinical practice.

## Introduction

The first 1000 days of life–a period beginning at conception and extending up to two years after birth–play a critical role in maternal, newborn, and child health (MNCH) and influence health trajectories and outcomes in subsequent life stages [1, 2]. This period provides a powerful window of opportunity for both pregnant women and new mothers with their babies to implement disease prevention and health promotion measures [3]. As pregnant women are more sensitive to external influences [4], seeking health information plays an important role in the development of health behaviors. For this reason, it is crucial that women receive accurate health information during the first 1000 days of life [5]. In this context, the literature shows that parents use digital media to learn about pregnancy [6] and their children’s health and development [7]. In addition, the restrictions imposed during the COVID-19 pandemic have encouraged the use of mobile health (mHealth) solutions, as they allow to overcome the barriers of traditional health services (e.g., economic barriers, time or space constraints), and women’s concerns about being in crowded spaces or waiting where there is an increased risk of becoming infected [8, 9]. Telehealth, or technology-enabled health care that includes health and health-related services delivered using digital communication and telecommunications technologies [10, 11], has increasingly come to the fore in this context. Despite its many devastating effects, the COVID-19 pandemic has indeed helped to accelerate and improve the implementation of telehealth worldwide, which has proven to be a helpful tool for uninterrupted care in times of physical distance [12]. In spite of the many difficulties faced by clinicians and healthcare institutions in adopting telehealth, it is rapidly evolving in clinical practice with the aim of integrating rather than replacing current standard of care. The adoption and use of telehealth have in fact been shown to improve the quality, accessibility, and affordability of healthcare services [13]. Similar benefits emerged from analyses examining opinions and attitudes of primary users (i.e. mothers and pregnant women) toward digitization of existing healthcare services in obstetrics and gynecology [14].

Many attempts have been made to introduce mHealth solutions aimed at supporting pregnant women and new mothers during the crucial period of the first 1000 days of life by harnessing the potential of digital tools to meet the growing diverse needs emerging at this stage of life [4, 7, 15]. Thus, women experiencing the life-changing period of childbearing have expressed a lack of adequate support at informational, psychological, experiential, and practical and material levels, highlighting heterogeneous needs that require different knowledge, resources, and skills to be met [16]. Despite these promising premises, several barriers to integrating mHealth solutions into clinical practice have been identified, particularly in relation to their validation. First, there is limited evidence of the effectiveness of using mHealth in promoting MNCH. For this reason, we felt it was essential to begin conducting rigorous studies aimed at designing (or co-designing with end-users), implementing, and subsequently evaluating telehealth interventions in this healthcare area [17–19]. Indeed, many available apps have been found to be unable to meet the needs and expectations of primary users and health professionals (i.e., secondary users) in terms of content validity [20, 21], functionalities, and technical features [21, 22]. Finally, most apps do not consider the continuum between pregnancy, birth, and postpartum and ultimately neglect the collective impact of the first 1000 days of life on women’s and children’s health [19, 21, 23].

Considering the shortcomings highlighted, the aim of this study was to explore the needs and expectations of hospital-based health professionals involved in the care of pregnant women and new mothers with their babies for a hypothetical app to support the first 1000 days of life. The goal of this research is to provide useful information for the development of an effective mHealth solution that can act as a link between the needs and expectations of end-users and the potential ability of the technological tool to meet them. Specifically, what information content, functionalities, and technical features of an mHealth app for the first 1000 days of life are most desirable to primary users (i.e., pregnant women and new mothers) from the perspective of secondary users (i.e., health professionals)? Furthermore, what do health professionals expect from the use of an mHealth app to support the first 1000 days?

## Materials and methods

### Study design and data collection

Between November 2021 and March 2022, all health professionals involved in maternal and child care, namely physicians and medical residents (staffed in gynecology, nursery/neonatology, and pediatrics), midwives, nurses, and support staff working or studying at the 136-bed Institute for Maternal and Child Health- IRCCS “Burlo Garofolo” in Trieste, Italy (henceforth referred to as “IRCCS Burlo Garofolo”), were invited to participate in this cross-sectional study by completing a questionnaire. Enrollment was voluntary ad participants were informed of the study purpose and were asked for a written informed consent to take part in our study. To ensure confidentiality, the consent form and completed questionnaire were linked with a random code. The study was approved by the Institutional Review Board of the IRCCS Burlo Garofolo (Code: IRB-BURLO 08/2021).

Given the complexity of healthcare during the first 1000 days of life [24], the selection of questionnaire items (i.e. variables to evaluate) used in this study was based on an analysis of the existing literature, and on the result of a guided interdisciplinary discussion [25] conducted by the expert panel in this research team (including specialists in gynecology and obstetrics and in public health, nurses, midwives, a psychologist, a sociologist, and an engineer, among others) on available recent findings, especially the main explored by mHealth research focused on healthcare promotion in pregnancy and in the postpartum period [21]. Within this process of analysis and synthesis, an essential theoretical premise as well as a crucial reference framework for the conceptualization and development of the questionnaire was the technical policy document prepared at the initiative of the Italian Ministry of Health containing a series of evidence-based recommendations for health-promoting interventions in the first 1000 days of life [2].

The questionnaire comprised 83 items related to the following six domains: pregnancy care and counselling (26 items), postnatal care and counselling for both mother and child (13 items), reminders and push notifications (4 items), notes and records (13 items), social support (4 items), and app technical features (23 items). The questionnaire items examined the importance that health professionals place on a range of content, functionalities, and technical features of a hypothetical mHealth app to support pregnant women and new mothers with their babies in the first 1000 days of life. For each item, participants gave their ratings on a 5-point Likert scale (0-not important at all; 1-of little importance; 2-of average importance; 3-very important; 4-absolutely essential). The 83-item questionnaire was supplemented by four additional questions (multiple-choice items on set categories and a free-text option) aimed at health professionals assessing the sources of information most frequently used by their patients (i.e. pregnant women and new mothers), the improvements health professionals expect to see from using a dedicated mHealth app to support the first 1000 days of life, the settings and situations in which pregnant women and new mothers typically receive information and advice, and the main barriers health professionals experience or perceive in communicating effectively with their patients. In addition, the following data were collected on the sociodemographic characteristics of health professionals: professional profile, working area, and years of work experience. Participants completed the survey using paper and pencil in approximately 15 minutes under the general supervision of a researcher who was available to provide clarification as needed.

### Data analysis

Descriptive statistics on the collected data were provided as percentages among tested and mean score per item. Predictors (i.e. independent variables related to professional profile, working area and years of work experience) and outcome variables (i.e., scored responses to questionnaire items) were explored by cross-tabulation and tested for correlation (Pearson’s r), independence (Chi2 test correcting with Fisher’s exact statistic of the categorical variables professional profile, working area and years of work experience), and normality distribution (Shapiro-Wilk normality test) of the outcomes variables. The significance for hypothesis was set at the 0.05 level. After exclusion of one item because of a typing error that mislead the respondent, the remaining 83 items were tested for missing values, and items with more than six missing values were excluded, as were observations with more than four missing values. The total of 64 missing values in the 82 items (0.5%) were filled using multivariate imputation via chained equations MICE function with five imputed records and ten maximum interactions. Ward hierarchical clustering method was used to classify respondents according to their response patterns. Various cluster numbers from two to eight were examined based on the ability to meaningfully describe response behavior in terms of the six questionnaire domains. The latter was defined as the average score of respondents for each questionnaire domain (i.e., the sum of the scores of all questions in a domain divided by the number of questions in that domain). The domain average score was plotted against the cluster grouping, and the final decision on the number of clusters was made by the authors’ judgment. Data were analyzed in R using the R packages: dplyr, ggplot2, Hmisc, mice, and pvclust [26].

## Results

During the study period, 145 questionnaires were collected by convenience sampling from hospital-based health professionals fulfilled the inclusion criteria for missing values. Physicians, medical residents, and nurses were evenly distributed between obstetrics/gynecology and pediatrics units (17:16, 23:20, and 10:14, respectively), whereas midwives (21/30, 70%) and support staff (11/15; 73%) were mainly from obstetrics/gynecology unit. The sociodemographic characteristics of survey participants are shown in Table 1.

**Table 1:** Socio-demographic characteristics of survey participants.

Respondent	Variable	Value n (%)
Health professionals (n = 145)	Professional profile	
	Physician	33 (23)
	Medical resident	43 (30)
	Midwife	30 (21)
	Nurse	24 (16)
	Support staff	15 (10)
	Working area	
	Obstetrics/gynecology	82 (57)
	Nursery/neonatology	15 (10)
	Pediatrics	48 (33)
	Years of work experience *(missing 4)*	
	< 3 years	29 (20)
	3–5 years	35 (24)
	> 5 years	77 (53)

When asked about the sources of information most frequently used by pregnant women/new mothers, health professionals cited social media (n = 120; 83%), followed by communities of practice (n = 102; 70%) and live communities (n = 96; 66%). Regarding the expectations related to the use of an mHealth app to support the first 1000 days of life, health professionals focused primarily on improved communication with patients (n = 100; 69%) and better preparation of pregnant women/new mothers (n = 88; 61%), while their expectations for a reduction in the time spent informing patients were lower (n = 51; 35%). The responses of the health professionals are shown in Table 2. Moreover, secondary users reported that settings and situations routinely involving the exchange of information with patients included individual counseling sessions (n = 127; 88%), prenatal classes (n = 107; 74%), and ultrasound examinations (n = 91; 63%) and prenatal screening procedures (n = 52; 36%). Three out of four health professional profiles (i.e., physicians, midwives, and support staff) mentioned three of these educational opportunities, while medical residents and nurses referred to only two of them on average. Health professionals cited the spread of fake news (n = 123; 85%), language difficulties (n = 100; 69%), and low patient education levels (n = 57; 39%) as major barriers to information sharing with hospital users in the context of professional practice.

**Table 2:** Summary of secondary users’ responses on the sources of information most frequently used by their patients and the improvements expected from using an mHealth app to support the first 1000 days of life.

Variable	Health professionals (n = 145)
Sources of information most used by pregnant patients/new mothers*; n (%)	
Social media	120 (83)
Communities of practice^§^	102 (70)
Live communities^§§^	96 (66)
Digital communication tools	64 (44)
Certificated information sources (e.g., institutional websites)	15 (10)
Others	0 (0)
Expected improvements from the use of a dedicated mHealth app*; n (%)	
Improved communication between pregnant women/new mothers/couples and health professionals	100 (69)
Increased preparation of pregnant women/new mothers	88 (61)
Optimization of time spent providing information by health professionals	51 (35)
Use of a common code	42 (29)
Others	0 (0)

*multiple answers allowed;

^§^ informal entities comprising groups of people who share an interest in something that they are capable of doing and who interact regularly to learn how to do it better [27], such as training groups and peer groups;

^§§^ groups of people interested in a specific topic who communicate with each other via a telematic network, such as the Internet or telephone networks, e.g., online platforms, websites, blogs, and forums

Regarding the desirable information content of an mHealth app for the first 1000 days of life, the two content items that health professionals considered most important were general information about health during pregnancy (absolutely essential or very important for 133; 92%) and information about infections that can occur during pregnancy (absolutely essential or very important for 132; 91%). Three health professionals suggested adding content about sexuality during pregnancy and after birth to the proposed app. Less well rated were some functionalities of the app, such as integration with social networks (little or not important at all for 73; 50%), the ability to schedule appointments (little or not important at all for 68; 47%) or record reminders for routine activities (little or not important at all for 64; 44%), the possibility to keep a sleep diary for the mother (little or not important at all for 63; 43%) and the newborn (little or not important at all for 61; 42%), and, finally, the possibility to provide users with social mechanisms to interact with peers (little or not important at all for 49; 34%). The full list of content, functionalities, and technical features and their ratings by health professionals is shown in Table 3.

**Table 3:** Ratings from health professionals regarding the contents, functionalities, and technical features of the proposed app.

Domain	Item	Health professionals (N = 145)										
		Mean	not important at all		of little importance		of average importance		very important		absolutely essential	
			n	%	n	%	n	%	n	%	n	%
Pregnancy care and counselling	General information about pregnancy (e.g., nutrition/weight gain, fitness, oral health, travel health)	3.54	1	0.7	0	0.0	11	7.6	40	27.6	93	64.1
	Information about drugs that can be taken during pregnancy	3.36	2	1.4	9	6.2	12	8.3	34	23.4	88	60.7
	Information about vitamin supplement during pregnancy	2.82	2	1.4	14	9.7	34	23.4	53	36.6	42	29.0
	A list of free-of-charge and upon payment examinations to b carried out during pregnancy	3.41	0	0.0	4	2.8	13	9.0	47	32.4	81	55.9
	Information about infections that can occur during pregnancy (e.g., toxoplasmosis, cytomegalovirus)	3.56	1	0.7	2	1.4	10	6.9	34	23.4	98	67.6
	Information about the immunizations that the mother needs to receive during pregnancy	3.41	0	0.0	4	2.8	13	9.0	47	32.4	81	55.9
	Information about voluntary pregnancy interruption	3.21	3	2.1	5	3.4	24	16.6	40	27.6	73	50.3
	Information about spontaneous miscarriage	3.12	3	2.1	3	2.1	31	21.4	44	30.3	64	44.1
	Information about violence/abuse during pregnancy	3.14	4	2.8	10	6.9	17	11.7	44	30.3	70	48.3
	Information about possible problems related to pregnancy	3.17	3	2.1	3	2.1	26	17.9	48	33.1	65	44.8
	Information on fetal development	3.20	1	0.7	6	4.1	22	15.2	50	34.5	66	45.5
	Information about fetal movements	3.38	3	2.1	5	3.4	10	6.9	43	29.7	84	57.9
	Information about prevention measures during pregnancy (e.g., home accidents, car accidents)	3.17	1	0.7	7	4.8	28	19.3	39	26.9	70	48.3
	Information about labour	3.41	4	2.8	3	2.1	7	4.8	46	31.7	85	58.6
	Information about delivery (e.g., epidural anaesthesia, caesarean section)	3.45	1	0.7	5	3.4	3	2.1	55	37.9	80	55.9
	Information about cord blood donation	2.70	5	3.4	14	9.7	35	24.1	56	38.6	35	24.1
	Information about pre-delivery courses	3.17	3	2.1	4	2.8	23	15.9	50	34.5	65	44.8
	Information about in-hospital stay for mother and newborn at the time of delivery	2.90	2	1.4	6	4.1	37	25.5	60	41.4	40	27.6
	Information about the woman’s rights during pregnancy (e.g., at work, at school, economical support)	3.32	2	1.4	2	1.4	20	13.8	44	30.3	77	53.1
	Information on prenatal risks and life-threatening conditions for both the mother and the foetus during pregnancy	3.28	1	0.7	4	2.8	19	13.1	50	34.5	71	49.0
	Information about maternal physiological and metabolic changes occurring during pregnancy	2.97	1	0.7	10	6.9	28	19.3	59	40.7	47	32.4
	Information about maternal or child services accessibility and contacts	3.19	0	0.0	3	2.1	33	22.8	42	29.0	67	46.2
	Information about available prenatal diagnostic tests	3.28	1	0.7	4	2.8	12	8.3	65	44.8	63	43.4
	Information about physical exercises and workouts for women during pregnancy	2.89	2	1.4	9	6.2	30	20.7	66	45.5	38	26.2
	Month/trimester-related tips for pregnant women	3.07	2	1.4	6	4.1	23	15.9	63	43.4	51	35.2
	A list of essentials for the hospital luggage	2.19	11	7.6	32	22.1	43	29.7	36	24.8	23	15.9
Postnatal care and counselling for both mother and child	A list of essentials for the first welcome of the mother and baby at home	2.83	5	3.4	12	8.3	37	25.5	40	27.6	51	35.2
	Information about maternal physiological and metabolic changes occurring during the postpartum period	3.01	2	1.4	8	5.5	34	23.4	43	29.7	58	40.0
	Information about manifest neonatal complications and warning signs	3.51	2	1.4	4	2.8	9	6.2	33	22.8	97	66.9
	Information about postpartum mental disorders, such as postpartum depression and baby blues (e.g., symptoms and coping strategies)	3.39	3	2.1	3	2.1	12	8.3	43	29.7	83	57.9
	Tips for the postpartum recovery process	3.10	2	1.4	3	2.1	26	17.9	62	42.8	52	35.9
	Information about breastfeeding practices	3.29	3	2.1	6	4.1	12	8.3	49	33.8	75	51.7
	Information on mother’s nutrition after childbirth and during breastfeeding	2.97	1	0.7	6	4.1	35	24.1	58	40.0	45	31.0
	Information about drugs compatible with breastfeeding	3.28	1	0.7	5	3.4	16	11.0	54	37.2	69	47.6
	Practical tips on how to take care of the newborn (e.g., hygiene, diapers’ changing, and burping)	3.19	2	1.4	0	0.0	27	18.6	55	37.9	61	42.1
	Information about neonatal screening procedures	3.14	3	2.1	3	2.1	24	16.6	55	37.9	60	41.4
	Information about the immunizations that mothers or newborns need during the first 1000 days	3.35	1	0.7	4	2.8	18	12.4	42	29.0	80	55.2
	Information about risk prevention measures regarding the newborn	3.20	1	0.7	5	3.4	29	20.0	39	26.9	71	49.0
	Information about methods for postpartum family planning and birth spacing	2.98	2	1.4	10	6.9	26	17.9	58	40.0	49	33.8
Reminders and push notifications	App’s ability to send push notification reminders when the pregnancy month/trimester begins	2.29	14	9.7	21	14.5	44	30.3	41	28.3	25	17.2
	App’s ability to schedule reminders for routine activities (e.g., drinking, diapering, feeding, pumping, and sleeping)	1.66	33	22.8	35	24.1	39	26.9	25	17.2	13	9.0
	App’s ability to change reminders and notifications settings	2.44	6	4.1	23	15.9	45	31.0	43	29.7	28	19.3
Notes and records	App’s ability to record the latest period date or the expected delivery date	2.54	9	6.2	19	13.1	36	24.8	46	31.7	35	24.1
	App’s ability to change the expected delivery date following medical re-evaluation	2.66	8	5.5	16	11.0	32	22.1	51	35.2	38	26.2
	App’s ability to record physiological values of the mother (e.g., pressure, temperature, and mood)	2.44	10	6.9	19	13.1	37	25.5	55	37.9	24	16.6
	App’s ability to record contractions or kicks	2.28	15	10.3	27	18.6	34	23.4	40	27.6	29	20.0
	App’s ability to record routine activities of the mother or the newborn (e.g., drinking, steps, diapers changes, bottle feeding, and sleeping patterns/times)	1.73	27	18.6	37	25.5	42	29.0	26	17.9	13	9.0
	App’s ability to record the medical care the mother or the newborn has received (eg, medications and vaccination shots)	2.63	7	4.8	18	12.4	32	22.1	52	35.9	36	24.8
	App’s ability to track the newborn’s developmental milestones	2.31	13	9.0	21	14.5	41	28.3	48	33.1	22	15.2
	App’s ability to record anthropometric measurements of the fetus (e.g., height, weight, and head circumference)	2.41	14	9.7	16	11.0	40	27.6	47	32.4	28	19.3
	App’s ability to record anthropometric measurements of the newborn (e.g., height, weight, and head circumference)	2.37	10	6.9	20	13.8	45	31.0	46	31.7	24	16.6
	App’s ability to record measurements of the mother’s weight at baseline and during pregnancy	2.41	12	8.3	15	10.3	41	28.3	56	38.6	21	14.5
	App’s ability to record measurements of the mother’s weight in the postnatal period	1.98	17	11.7	32	22.1	46	31.7	37	25.5	13	9.0
	App’s ability to create a sleep diary for the mother	1.74	30	20.7	33	22.8	40	27.6	29	20.0	13	9.0
	App’s ability to create a sleep diary for the newborn	1.83	26	17.9	35	24.1	40	27.6	26	17.9	18	12.4
Social support	Integration of the app with social networks (e.g., Facebook and Twitter)	1.51	40	27.6	33	22.8	39	26.9	24	16.6	9	6.2
	Presence of a FAQ (frequently asked questions) page in the app	2.48	10	6.9	18	12.4	39	26.9	49	33.8	29	20.0
	Presence of social mechanisms allowing users to interact with each other and share experiences (e.g., community, forum, and chat)	1.85	26	17.9	23	15.9	53	36.6	33	22.8	10	6.9
	Presence of social mechanisms allowing users to interact with health care staff (e.g., community, forum, and chat)	2.44	10	6.9	19	13.1	40	27.6	49	33.8	27	18.6
App technical features	Authentication request to the user	2.59	10	6.9	24	16.6	29	20.0	34	23.4	48	33.1
	Presence of a privacy policy in the app	2.77	7	4.8	17	11.7	31	21.4	37	25.5	53	36.6
	If present, availability of privacy policy properly written in Italian	2.72	7	4.8	17	11.7	34	23.4	38	26.2	49	33.8
	Ability for the user to access all app contents for free (without any payment)	3.31	3	2.1	10	6.9	16	11.0	26	17.9	90	62.1
	Access to full app usage based on specific inclusion criteria (e.g., national health service card, place of living, and certification by a health professional)	2.35	14	9.7	34	23.4	23	15.9	35	24.1	39	26.9
	Requirement for the user to sign an informed consent to use the app	2.40	13	9.0	29	20.0	27	18.6	39	26.9	37	25.5
	Presence of references about the provided contents	2.68	7	4.8	16	11.0	33	22.8	49	33.8	40	27.6
	Presence of a glossary of the most used medical terms	2.89	6	4.1	9	6.2	31	21.4	48	33.1	51	35.2
	Declaration (through provision of references) of the scientific responsibility of the contents provided by the app	2.44	8	5.5	22	15.2	37	25.5	54	37.2	24	16.6
	Possibility to back-up/restore data within the app	2.37	11	7.6	22	15.2	40	27.6	46	31.7	26	17.9
	Possibility to download data collected through the app	2.60	8	5.5	13	9.0	41	28.3	50	34.5	33	22.8
	Presence of a multilanguage support	3.37	1	0.7	6	4.1	21	14.5	27	18.6	90	62.1
	App’s ability to geolocate the user to provide more detailed information	1.77	30	20.7	30	20.7	40	27.6	34	23.4	11	7.6
	App’s ability to book visits, vaccinations, and checkups	3.09	5	3.4	10	6.9	18	12.4	46	31.7	66	45.5
	App’s ability to update user’s account preferences	2.74	5	3.4	17	11.7	30	20.7	52	35.9	41	28.3
	Use of a simple, informal, and friendly tone by the app	2.87	6	4.1	14	9.7	24	16.6	50	34.5	51	35.2
	App’s ability to adapt to screen orientation (both portrait and landscape)	2.59	8	5.5	22	15.2	37	25.5	33	22.8	45	31.0
	App’s ability to learn user’s preferences over time	2.15	18	12.4	27	18.6	41	28.3	33	22.8	26	17.9
	App’s ability to implement intuitive and predictable navigation patterns	3.14	3	2.1	9	6.2	22	15.2	41	28.3	70	48.3
	Presence of app contents validated by an institutional source (local, regional, or national)	2.99	3	2.1	12	8.3	27	18.6	45	31.0	58	40.0
	Presence of a certification of the app as a medical device according to Italian law	2.31	15	10.3	24	16.6	39	26.9	35	24.1	32	22.1
	App’s ability to provide contents through different ways (e.g., text, video, and audio)	2.68	7	4.8	15	10.3	34	23.4	50	34.5	39	26.9
	App’s ability to ask about user satisfaction	2.44	11	7.6	22	15.2	35	24.1	44	30.3	33	22.8

**Fig 1:**
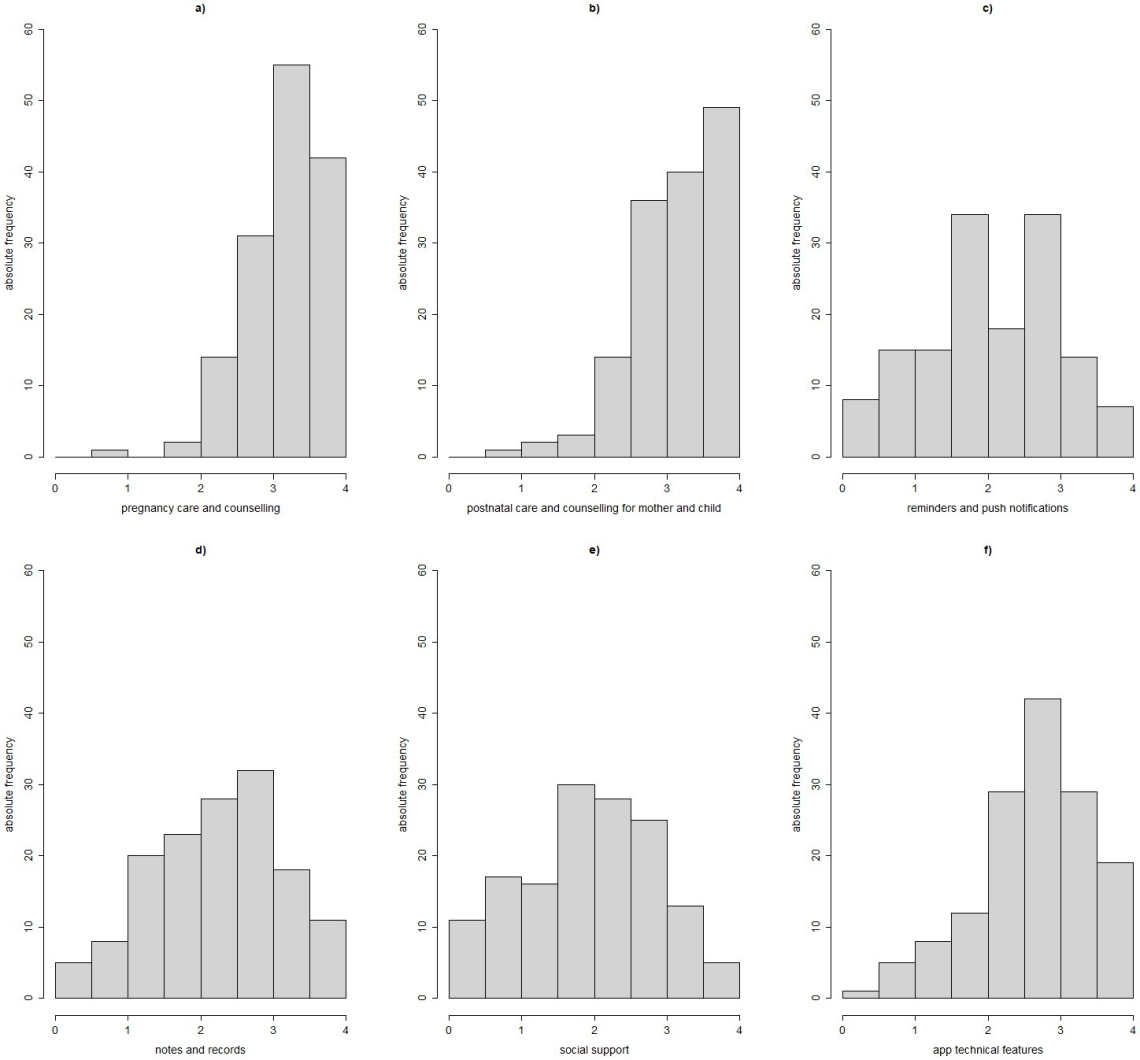
Distribution of health professionals’ opinions on the contents, functionalities, and technical features of an mHealth app to support the first 1000 days of life, grouped by the six questionnaire domains.

The distribution of average scores related to the domains of desirable content, functionalities, and technical features for the mHealth app from secondary users is shown in Fig 1. Overall, higher scores were provided to domains “pregnancy care and counselling”, “postnatal care and counselling for both mother and child”, and “app technical features” as shown by the left-skewed distributions of a), b) and f) plots in Fig 1.

**Fig 2:**
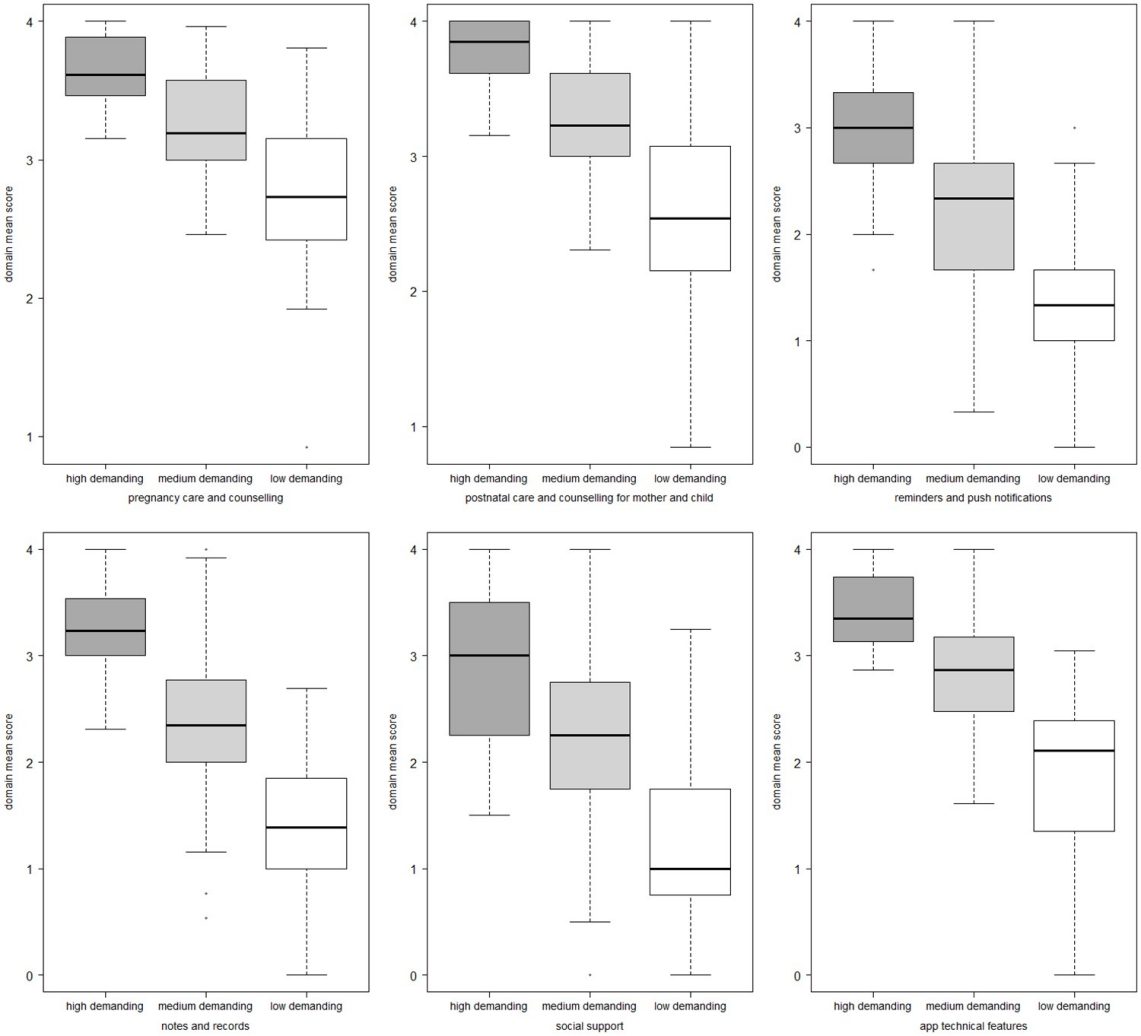
Distribution of health professionals’ responses by cluster (i.e., high, medium, and low demanding clusters) for each questionnaire domain.

Secondary users were grouped in three clusters according to their responses: medium demanding cluster included 63.4% (92/145) of all respondents, while high demanding and low demanding clusters represented 20.7% (30/145) and 15.9% (23/145) of them, respectively. The three clusters-cut of the cluster tree matched well with the ranges of the items, so that the respondents with high, medium and low scores were clearly identified, as shown in Fig 2. High demanding cluster narrowly grouped on very high average score (e.g., very important or absolutely essential) on the questions about pregnancy care and counselling, postnatal care and counselling for both mother and child, app technical features, and notes and records domains. Responses in medium demanding cluster also had high scores for the same four domains (median close to three), but their distribution was broader. Low demanding cluster was characterized by more skeptical attitudes toward all items, especially those related to the reminders and push notifications, notes and records, and social support domains.

Analyzing the composition of the three clusters, we found that high demanding cluster was evenly composed of the different groups of health professionals involved (21% physicians, 17% medical residents, 21% nurses, 14% midwives), with a slight predominance of support staff (27%). Medium demanding cluster had a higher proportion of medical residents (35%), a lower proportion of support staff (8%), and similar proportions of physicians (16%), nurses (18%), and midwives (23%). Finally, low demanding cluster was composed of half by physicians (36%) and medical residents (29%), while support staff again accounted for a very small proportion (2%); in this latter cluster, midwives and nurses each accounted for 17%.

**Fig 3:**
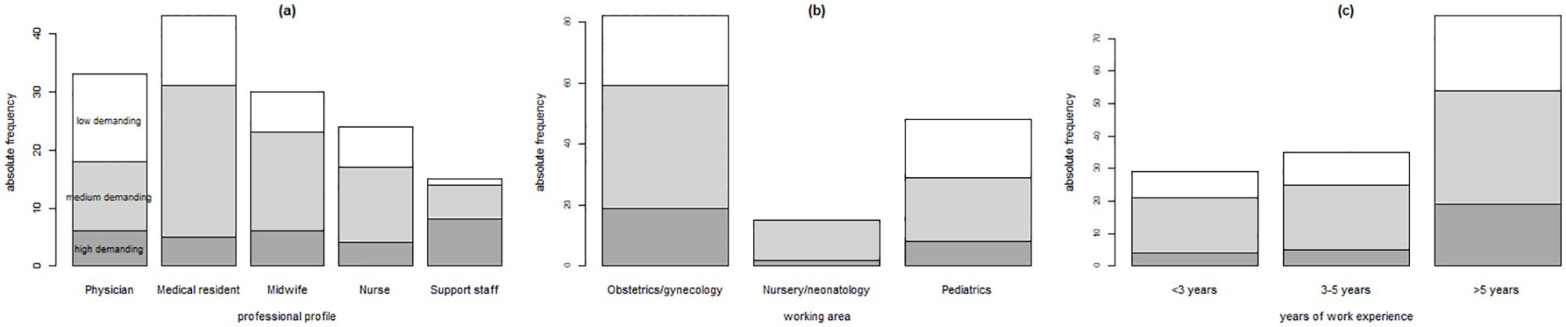
Distribution of clusters of health professionals (i.e., high, medium, and low demanding clusters) grouped by professional profile, working area, and years of work experience.

When analyzing the composition of each health professional group (i.e., physicians, medical residents, midwives, nurses, and support staff) in terms of overall domain scoring (i.e., belonging to high, medium or low demanding cluster) (see plot a) in Fig 3), most respondents (51%) were in medium demanding cluster (60% of medical residents, 57% of midwives, and 54% of nurses). The high demanding cluster included 53% of the total support staff which participated in the study. These differences were statistically significant (p = 0.02). In reference to the working area, as shown by plot b) in Fig 3, the 87% of the health professionals belonging to nursery/neonatology area, 49% from the obstetrics/gynecology area, and 44% of those from the pediatrics area were grouped in the medium demanding cluster. There were no professionals working in the nursery/neonatology area in low demanding cluster. The difference in the clusters composition by working area was statistically significant (p = 0.02). Health professionals with intermediate work experience (3–5 years) were more likely to belong to the medium demanding clusters (57%) (plot c) in Fig 3). These differences were not statistically significant.

## Discussion

Our study aimed to explore the needs and expectations of secondary users (i.e., hospital-based health professionals) for an mHealth app to support the first 1000 days of life. We found that health professionals generally acknowledge the importance of the proposed app content in the domains of pregnancy care and counselling and postnatal care and counselling for mother and child, as well as the technical features of the app. Nevertheless, health professionals expressed skepticism about several app functionalities in the domains of reminders and push notifications, notes and records, and social support. Based on the assumption that a participatory co-design approach is crucial for developing effective solutions, this study provides a comparative analysis of the evaluations of the proposed mHealth app made by different groups of health professionals and highlights points of contact and discrepancies between their perceptions. From this perspective, our research also provides a framework and guidance for the development and implementation of increasingly customized mHealth apps that meet the needs and expectations of secondary users in terms of content, functionalities, and technical features.

Proper information and education of patients and citizens are milestones of disease prevention and health promotion, and the importance of combating infodemic and fake news has been and continues to be a burning issue, especially in times such as the COVID-19 pandemic, which was characterized by an overabundance of information from dubious sources [28, 29]. Health professionals indicated that communities of practice are among the most important sources of information for pregnant women and new mothers (i.e., primary users). Indeed, this is consistent with the findings of a recent study by the authors [30] showing that expectant and new parents use communities of practice as their main source of information on pregnancy and postpartum period. The actual opportunity to benefit from these social interactions could be reconsidered, as well as the time spent on education during regular consultations and clinical or ultrasound examinations, especially at times of physical distance. Moreover, health professionals think it is unlikely that their patients are accustomed to consulting certified sources and instead believe that the sources of information they use most often are social media. This is in contrast to the authors’ recent findings that expectant and new parents are more likely to use certified sources (e.g., guidelines and service cards) than social media (e.g., Facebook and Twitter) when seeking information on pregnancy and postpartum period [30]. This inconsistency may be attributed to a knowledge gap among health professionals who are unaware of the information channels and tools used by their patients, or/and to patients’ uncertainty on what actually is a certified and reliable source of information. This is an open question that needs further investigation. In this sense, the role of high levels of health literacy and digital health literacy should not be neglected, as they have been shown to be effective resources for the proper use of health information and are also the strongest predictors of health status and well-being, ahead of income, employment status, education level, and race or ethnicity [31]. Anyway, this discrepancy between secondary users’ perceptions and primary users’ statements should trigger healthcare organizations and professionals to improve their support for pregnant women and new mothers (and, by extension, expectant and new parents) by promoting the knowledge and dissemination of institutional resources, i.e., reliable sources of information par excellence, among users. Consistent with these considerations, the availability of an institutional, certified, and multilingual mHealth app for maternal and child care focused on the first 1000 days of life could play a critical role in providing reliable, validated, and evidence-based information, with potential positive spillover effects on the development of disease-preventive and health-promoting behaviours by primary users. According to health professionals, such an app could first improve patient-professional communication and then increase preparedness among pregnant women and new mothers, which would likely have a positive impact on health outcomes. Primary users would be able to read and analyze the information provided by the mHealth app, depending on the amount of time available, and then discuss the content with health professionals at the earliest opportunity. In addition, an all-day digital tool can help women learn basic concepts and some specific terminology by improving their health literacy. Providing primary users with a common ground for discussion and mutual understanding can make communication with health professionals easier and more effective, and leaves more time for clarifying any doubts or addressing other patient needs. Concerning the content of the app, health professionals generally recognized the value of the proposed information and felt that content about health and possible high-risk infections during pregnancy was particularly important. The technical features of the proposed app were also generally appreciated by secondary users, who considered the presence of a multilingual support, the ability for the user to access all app content for free, the app ability to implement intuitive and predictable navigation patterns, and the app ability to book visits, immunizations and check-ups particularly important.

In terms of functionality, contrary to what theorized [21], health professionals did not seem very interested in using mHealth apps to integrate health services with social media and to support social interactions between patients. Health professionals tended to exclude this option from effective and desirable interactions with patients, perhaps arguing that the continuous exchange of health information and potential overuse of tracking features could lead to negative outcomes, which have already been reported in the form of an increase in false-positive triggers and unnecessary medical interventions [32]. This type of resistance could indicate the presence of a generation of health professionals or a system that is not yet ready to implement telehealth in practice. In this regard, research points to several factors at the individual, organizational, and contextual levels that influence the adoption of mHealth apps by health professionals. Among other factors, issues related to perceptions of increased workload and managing additional mHealth-related tasks have been cited as potential barriers to telehealth adoption [33, 34]. Other obstacles reported in the literature include the gap in health system design for such implementation, lack of organizational support, staff shortages, age of existing technical equipment, and lack of personal skills and expertise in this new area among healthcare workers [35, 36]. Concerning the health professionals participating in the present study, support staff, who tended to be concentrated in high demanding cluster, recognised the importance of certain aspects of the proposed app more than participants with other professional profiles. Conversely, medical residents who belonged mainly to medium and low demanding clusters were more sceptical about the usefulness of certain functionalities and technical features of the app, particularly in the domains of reminders and push notifications, notes and records, and social support. The difference in importance attached to the proposed app could be related to the different roles that support staff and medical residents occupy in patient care (e.g., in terms of tasks performed in daily practice), as well as the diverse responsibilities of these health professionals in communication and decision-making processes in care. Indeed, these issues may be related to the different perceptions of these two categories of health professionals regarding the ability of the proposed app to actually help them perform their tasks more efficiently and effectively [37], and ultimately the feasibility of rapid and effective adoption and integration of mHealth solutions into standard clinical practice with measurable benefits and improvements. Considering the challenges, but also the opportunities associated with the use of mHealth by healthcare professionals [33], mHealth solutions should not be seen as a substitute for direct human contacts and interpersonal relationships but as a useful supplementary tool to be applied alongside conventional services involving paper-based tools [14] and face-to-face information exchange for those who, for example, are less positive or skeptical about digitization or cannot afford advanced digital devices [34].

The mobile personal health record has already become the new de facto standard for mHealth [20], and legal, institutional, and systemic frameworks are needed for effective implementation of telehealth. The readiness of health professionals for this “revolution” seems to be quite high, as other studies have also shown [20, 38], but for the benefits of this paradigm shift to be quantified, methodologically rigorous measures need to be implemented and scientifically evaluated [20], including the use of tools to study the economic, institutional, and social impact of the application of telehealth and its integration into the healthcare system [39].

### Study limitations

This study has some limitations that may limit results generalization and must be accounted for properly interpret our findings. First, the reference population does not include primary and community healthcare workers, such as midwives and psychologists at local health unit family centers or general paediatricians yet involved in health promotion in the first 1000 days of life. All the health professionals invited to participate in the study came indeed from the IRCCS Burlo Garofolo, thus representing only the hospital setting. Accordingly, our research should not be considered a prevalence study because of sampling by convenience and not proportional to the regional distribution of health care workers. However, given the international excellence that the IRCCS Burlo Garofolo has achieved in the field of maternal and child health, we consider a representative context as well as a privileged observatory, worthy of study, since it serves a large regional and national catchment area, thus dealing with a wide and heterogeneous population sample. Second, as for most of studies based on voluntary participation and involving the collection of self-report measures, health professionals enrolled may be biased for reasons of social desirability. Moreover, given the multiplicity and heterogeneity of areas of preventive, protective, and curative intervention in the first 1000 days of life [2], the questionnaire administered to participants addresses only some of the most important elements characterizing this phase of life, thus not covering all its aspects. However, as previously indicated, the topics explored in the questionnaire were identified on the basis of the existing literature [2, 40] and focus on pregnancy and the early postpartum period. Moreover, as the questionnaire also included items aimed to evaluate health professionals’ opinions on the functionalities and technical features of the proposed app, an overall simplification of the topics related to the first 1000 days was considered necessary to avoid making the questionnaire too long for the participants.

In conclusion, our findings regarding health professionals’ to needs and expectations of an mHealth app for the first 1000 days of life result from a participatory co-design approach characterized by the involvement of end-user in the process of developing innovative solutions. In this regard, the evidence that emerged from our research can provide food for thought for both healthcare organizations and technology providers, the synergy of which is essential for the development of tools and services to support health close to the user. Overall, the evaluation conducted in our study shows the general agreement of secondary users with the proposed app, indicating good conditions for their participation in innovative telehealth interventions in MCNH. However, many efforts still need to be made at the systemic level to make things work, including the commitment of healthcare organizations to improve information, training, culture, and organizational processes to effectively integrate and implement telehealth into standard health care practice.

## Abbreviations

mHealth: mobile health
MNCH: maternal, newborn, and child health
